# Modulating Microglial Cells for Promoting Brain Recovery and Repair

**DOI:** 10.3389/fncel.2020.627987

**Published:** 2021-01-11

**Authors:** Dirk M. Hermann, Matthias Gunzer

**Affiliations:** ^1^Department of Neurology, University of Duisburg-Essen, University Hospital Essen, Essen, Germany; ^2^Institute of Experimental Immunology and Imaging, University of Duisburg-Essen, University Hospital Essen, Essen, Germany

**Keywords:** blood-derived immune cell, brain injury, brain ischemia, neuroimmunology, neurodegeneration, neuroplasticity, neurorepair

## Abstract

Representing the brain’s innate immune cells that interact vividly with blood-derived immune cells and brain parenchymal cells, microglia set the stage for successful brain remodeling and repair in the aftermath of brain damage. With the development of pharmacological colony-stimulating factor-1 receptor inhibitors, which allow inhibiting or depleting microglial cells, and of transgenic mice, allowing the inducible depletion of microglial cells, experimental tools have become available for studying roles of microglia in neurodegenerative and neurorestorative processes. These models open fundamental insights into roles of microglia in controlling synaptic plasticity in the healthy and the injured brain. Acting as a switch from injury to repair, microglial cells might open opportunities for promoting neurological recovery in human patients upon brain injury.

Immune responses play a central role in modulating brain injury and recovery postinjury (Anrather and Iadecola, 2016; Jayaraj et al., 2019). In the injured brain, complex cellular and molecular mechanisms are triggered, including the release of inflammatory cytokines and alarmins by damaged cells (Bianchi, 2007; Roth et al., 2018), glial activation (Neumann et al., 2015; Manrique-Castano et al., 2020), and the brain invasion of leukocytes belonging to the innate and adaptive immune systems (Gelderblom et al., 2009; Neumann et al., 2015). Immune cell trafficking across the blood–brain barrier is mediated by adhesion molecules on cerebral endothelial cells including intercellular adhesion molecule-1 and vascular cell adhesion molecule-1 (Lopes Pinheiro et al., 2016). In the injured brain, both peripheral leukocytes and resident microglia have been shown to accumulate in evolving brain lesions (Neumann et al., 2015; Perez-de-Puig et al., 2015).

Microglial cells, which are the brain’s innate immune cells, vividly interact with brain-invading leukocytes in the injured brain (Neumann et al., 2006, 2008, 2015), controlling their brain access and activity. Microglial cells also communicate with brain endothelial cells, maintaining the integrity of the brain microvasculature, and, specifically, the blood–brain barrier, as well as immune cell access to the injured brain (Dudvarski Stankovic et al., 2016). Microvascular protection by microglia is enabled by direct effects of microglia on endothelial cells, e.g., by inducing their phagocytosis or stabilizing endothelial cells by secretion of vascular endothelial growth factor (Dudvarski Stankovic et al., 2016). In organotypic brain slices *ex vivo* and experimental models of ischemic stroke *in vivo*, activated microglial cells were shown to engulf and phagocytose leukocytes (Neumann et al., 2008, 2018; Otxoa-de-Amezaga et al., 2019).

These dynamic responses of microglial cells in the injured brain were identified following advances in two fields, namely, in: (a) brain imaging and image analysis; and (b) pharmacological microglial deactivation and/or depletion. Brain imaging techniques facilitating the analysis of microglial cells are two-photon microscopy, which allows real-time imaging of cell physiology and pathology *in vivo* in the injured brain (Davalos et al., 2005; Neumann et al., 2015), and confocal microscopy combined with morphological image analysis, which *via* the segmentation, skeletonization, and three-dimensional reconstruction of microglial cells allows the evaluation of microglial activation in responses to injury and therapy (Heindl et al., 2018; Manrique-Castano et al., 2020). Innovations of brain imaging greatly promoted progress in our understanding of the contribution of microglial cells to brain recovery processes.

With the emergence of pharmacological colony-stimulating factor-1 receptor (CSF1R) inhibitors, which allow inhibiting and depleting microglial cells (Elmore et al., 2014; Waisman et al., 2015; Olmos-Alonso et al., 2016) and of the CX3CR1-CreER-R26iDTR mouse, in which microglia depletion can efficiently be induced by diphtheria toxin delivery (Parkhurst et al., 2013; Waisman et al., 2015), experimental tools have become available for assessing the role of microglial cells in neurodegenerative processes. Microglial depletion studies using a CSF1R inhibitor revealed that reactive microglia efficiently eliminate leukocytes from ischemic brain tissue (Otxoa-de-Amezaga et al., 2019). Microglia deactivation and depletion by long-term treatment with the CSF1R inhibitor increased brain leukocyte numbers, and microglial depletion enlarged brain infarcts (Otxoa-de-Amezaga et al., 2019). The combined evidence of these studies revealed that upon brain injury microglia set the stage for successful brain remodeling and repair.

Following these seminal works, our understanding of the role of microglial cells in brain remodeling and repair has strongly expanded in the last 2 years. Studies recently published in *Frontiers of Cellular Neuroscience* revealed how microglial cells push the balance toward remodeling and repair upon brain injury (Bernardino et al., 2020). Particularly noteworthy is the description of exosomes (Vaz et al., 2019), microparticles (Grimaldi et al., 2019), and secreted growth factors (Fuentes-Santamaría et al., 2019; Myhre et al., 2019; Wlodarczyk et al., 2019) as mediators of neuronal recovery induced by microglial cells. The role of microglia in controlling neurotransmission (Fuentes-Santamaría et al., 2019), neuronal myelination (Wlodarczyk et al., 2019), and synaptic plasticity (Gunner et al., 2019; Fuentes-Santamaría et al., 2019; Nguyen et al., 2020) was outlined. Further studies evaluated age and sex factors influencing microglial responses (Lively et al., 2018). These findings exemplify that manipulation of a distinct cell type allows modulating recovery processes in a clinically meaningful way.

Many open questions remain, e.g., with respect to: (a) bystanders of the induced degeneration of microglial cells in the living brain; (b) side effects of microglial deactivation or depletion on blood-derived immune cells, specifically of monocytes, which also carry CSF1R; and (c) the rapid repopulation of microglia following genetic or pharmacological microglia depletion. Some aspects of effects of microglial cells on neuronal and, specifically, synaptic plasticity still remain unresolved. These questions include whether or to which degree synaptic plasticity is influenced by synaptic pruning or nonphagocytic processes (Cheadle et al., 2020). Acting as a switch from injury to repair, microglial cells potently influence neurological recovery processes. Whether this strategy holds its promises in human patients still has to be shown.

## Data Availability Statement

Requests to access these datasets should be directed to dirk.hermann@uk-essen.de.

## Author Contributions

DH wrote the draft. Both authors revised and finalized it. All authors contributed to the article and approved the submitted version.

## Conflict of Interest

The authors declare that the research was conducted in the absence of any commercial or financial relationships that could be construed as a potential conflict of interest.
